# Extraction of the Structural Properties of Skin Tissue via Diffuse Reflectance Spectroscopy: An Inverse Methodology

**DOI:** 10.3390/s21113745

**Published:** 2021-05-28

**Authors:** Bin Chen, Yong Zhang, Shang Gao, Dong Li

**Affiliations:** State Key Laboratory of Multiphase Flow in Power Engineering, Xi’an Jiaotong University, Xi’an 710049, China; zhangyong2012@stu.xjtu.edu.cn (Y.Z.); gaoshang96@stu.xjtu.edu.cn (S.G.); lidong@mail.xjtu.edu.cn (D.L.)

**Keywords:** diffuse reflectance spectroscopy, inverse methodology, vascular dermatosis, skin phantom, laser speckle imaging

## Abstract

For the laser treatment of vascular dermatosis, the blood vessel morphology and depth in skin tissue is essential to achieve personalized intelligent therapy. The morphology can be obtained from the laser speckle imaging, and vessel depth was extracted by an inverse methodology based on diffuse reflectance spectrum. With optimized spot size of 0.5 mm and known optical properties, the proposed method was experimentally validated via the spectral measurement of microcapillary with known size and depth embedded in an epoxy resin-based skin phantom. Results prove that vessel depth can be extracted with an average relative error of 5%, thereby providing the foundation for a personalized, precise, and intelligent laser treatment of vascular dermatosis.

## 1. Introduction

As a typical congenital vascular dermatosis, port wine stain (PWS) birthmarks can negatively affect the physical and mental well-being of individuals because 90% of these marks appear on the face and neck [1]. PWS has been demonstrated to be curable via laser-mediated therapies based on selective photothermolysis [2]. However, the therapeutic effect of the laser on PWS is still far from satisfactory due to the complexity of the malformed capillaries [3], and the cure rate has remained below 20% for nearly 10 years via pulsed dye laser (585/595 nm) [4]. Recently, multipulse Nd:YAG laser (1064 nm) has demonstrated a preferable potential even for resistant PWS [5]. Nevertheless, the selection of laser parameters (e.g., frequency, fluence, and pulse number) lacks quantitative guidance. Similar problems exist in nearly all types of vascular dermatosis primarily due to experience-dependent treatments.

In the clinical laser treatment of PWS, non-invasive, fast and simple evaluation is required to improve the therapy efficacy. There are related non-invasive diagnostic technologies, including video microscopes and other imaging technologies based on charge coupled device (CCD) [6] and perfusion imaging technologies, such as laser Doppler imaging (LDI) [7], laser speckle imaging (LSI) [8], optical coherence tomography (OCT) [9], optical Doppler tomography (ODT) [10], photothermal radiometry (PTR) [11], photoacoustic (PA) [12], etc. Although we have investigated the influence of laser parameters (wavelength, pulse width, laser energy) [13] on the treatment effect, the monitoring and extraction of the structural features of the lesion still needs to be solved urgently.

**Figure 1:**
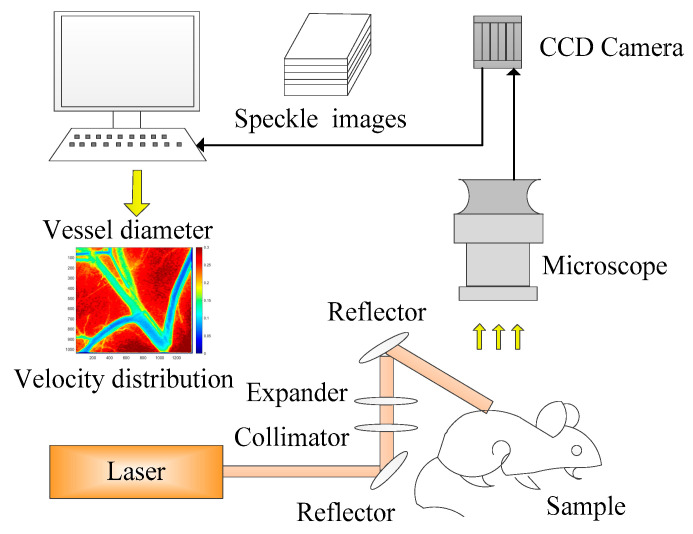
Schematic of laser speckle imaging experimental system.

Skin tissue consists of epidermis and dermis, with PWS vessels buried in dermis with diameter of 30~300 μm and depth of 200~800 μm [14]. During the exposure of multi-pulse laser irradiation, thrombus could be formed. According to our previous study, thrombus formation that completely occludes the vessel lumen has been proven as the prerequisite to achieve a desirable clinical end point (i.e., thread-like appearance) [15]. Based on our in-house integral moving particle semi-implicit (MPS) model to simulate the laser-induced blood coagulation [16], the optimal parameters of multi-pulse Nd:YAG laser can be theoretically recommended for different blood vessel size and depth, as shown in Figure 1. In other words, once blood vessel size and depth are known, personalized, precise, and intelligent laser treatment of vascular dermatosis can be achieved.

However, a problem still lies in detecting the subtle structure of skin tissue by available imaging techniques [17,18]. Ultrasound, computed tomography imaging, and magnetic resonance imaging systems can only locate large blood vessels with diameters ranging from 300 µm to 1000 µm (or more) [19,20,21]. The latest optical coherence tomography technology is effective [22], but it is expensive and cannot reflect information about blood flow and thrombosis, which is critical for the treatment of PWS. Laser speckle imaging (LSI) [5] can get blood vessel morphology and blood flow, but the depth of micro vessel cannot be extracted. Bjorgan et al. [23] used hyperspectral real-time processing and wavelet processing to enhance the contrast of the blood vessels in the tissue image, and they are the first to obtain the oxygen content and depth in the blood vessels through the inverse diffusion model.

Recently, considerable effort has been devoted to spectrometry-based imaging techniques, particularly diffuse reflectance spectroscopy (DRS) in the ultraviolet–visible (UV–VIS) spectrum [24]. This technique is advantageous because it is cost-effective, fast, nondestructive, and quantitative. The optical properties of biological tissues are the natural reflection of their geometric structure and physiological state. When a light beam with a certain wavelength irradiates onto biological tissues, the reflectance and transmittance spectra are formed by a portion of the scattered light escaping from the tissues. The optical properties of tissues can be obtained by measuring the reflectance spectrum. Then, tissue structure and physiological characteristics can be further deduced, which can provide guidance for evaluating medical effects, implementing histopathological and physiological diagnoses, and even for detecting the morphological changes of pathological tissues [25]. Potential and existing clinical applications of DRS rely on tissue diagnostics, including the monitoring of tissue oxygenation, tissue structure, cancer detection, and tissue response to laser therapies [25,26]. In the laser dermatology (e.g., PWS), the thickness of target tissues typically has the same order of magnitude as the detection depth of DRS, which may potentially facilitate the extraction of the structural parameters of the target blood vessels through the DRS analysis of skin tissues.

As mentioned earlier, the diameter and depth of the target PWS blood vessel are the key factors that determine the laser treatment parameters. One-to-one correspondence exists between the DRS and the combination of vessel depth and diameter, which can be proved by the Monte Carlo (MC) simulations of light propagation in a typical PWS tissue model [19]. Therefore, a unique combination of vessel depth and diameter can be ideally determined based on a known tissue reflectance by using the inverse radiation method. If blood vessel diameter can be obtained using laser speckle imaging [20], the inverse extraction of vessel depth will become a definite solution.

Available inverse approaches for detecting structure properties from a spectrum with known optical properties include the inverse adding–doubling (IAD) method [27,28] and inverse MC (IMC) method [29,30]. On the basis of the adding–doubling method, in which reflectance and transmittance are calculated by repeatedly doubling the initial tissue layer until the real thickness of the tissue domain is reached, Prahl et al. [28] developed the famous IAD method, which can provide a good prediction of the optical properties of bio-tissue model. However, IAD can only be implemented on a simple structure (i.e., 1D tissue model), in which the illumination diameter (spot size of laser) should be sufficiently small compared with the sample size (the influence of the spot size will be discussed in Section 3.2). Moreover, IAD can only provide a rough estimation of optical properties. By contrast, IMC is applicable to 3D cases over wide spectral bands due to the flexibility and powerful convergence of the MC method. However, numerous photons and multiple iterations restrict the real-time application of IMC. Taking the IAD result as the initial value for FMC simulation, computation can be accelerated with the reservation of 3D applicability.

The motivation of the current work is to gain an intuitive understanding of the relationship between DRS and the structure skin tissues, thereby determining blood vessel depth from the measured spectroscopic data with known vessel size from the laser speckle imaging. This methodology can be called the DRS-based inverse method. To achieve this objective, a theoretical model for extracting tissue structure was presented by integrating the measured reflectance spectrum and the inverse method within a wavelength range of 300 nm to 800 nm, which covered the window of laser therapy for PWS [31]. Quantitative in vivo experiment is difficult to conduct due to the inhomogeneity, uncertainty, and individual differences of biological tissues. Thus, a skin phantom with given blood vessel diameter and depth for characterizing PWS skin tissues was prepared to validate the theoretical model through the measurement of diffuse reflectance and transmittance via a high-precision integrating sphere. Section 2 describes the mathematical model of the DRS-based inverse method, along with the preparation and validation of the skin phantom. Section 3 presents the experimental and numerical studies of depth-related spectral signals. Conclusions are drawn in Section 4.

## 2. Method and Materials

### 2.1. Estimation of Blood Vessel Diameter Via LSCI

The ability to monitor blood flow and vessel diameter by LSCI was proved by in vivo animal experiment. A schematic of the LSCI experimental setup is shown in Figure 1. A collimated beam from a coherent laser emitted diode (Lambda Beam, RGB Photonics, Kelheim, Germany) with a wavelength of 808 nm, and maximum output power of 150 mW was expanded by the lens group to illuminate the dorsal skin chamber model. The illuminated region was recorded by a CCD camera (INFINITY3-1, Lumenera, Ottawa, ON, Canada) with a resolution of 1392 × 1040 pixels and a frame rate of 15 fps and was attached to a stereo microscope (M205A, Leica, Germany) with a maximum magnification of ×7.8 and a resolution of 2 mm. The CCD exposure time was set according to the diode laser power.

**Figure 2:**
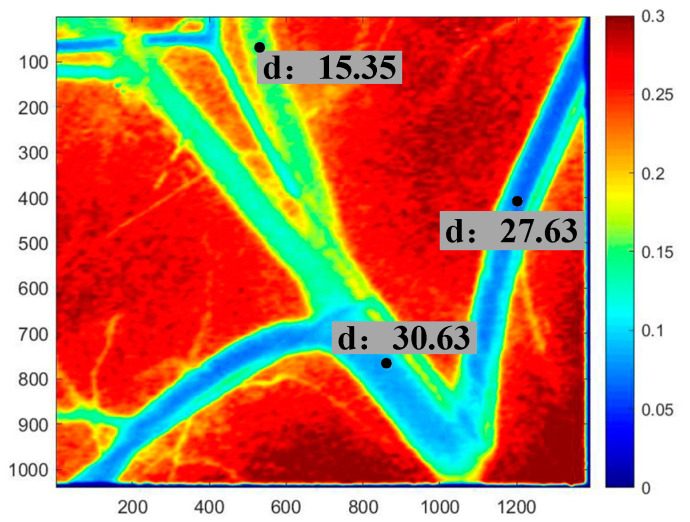
Diameter (μm) and velocity distribution (ms) obtained from speckle imaging.

Via the above LSCI system, diameter of blood vessel can be obtained from the speckle image, as shown in Figure 2. Through the speckle contrast method, blood flow velocity can be obtained. The lower the contrast value, the faster the flow velocity. Therefore, the blood vessel area and the tissue area can be distinguished, and next we will focus on the extraction of blood vessel depth.

### 2.2. Skin Phantom Preparation and Spectral Measurement

For real skin tissues, depth of blood vessel is difficult to control for the quantitative analysis of DRS. By contrast, skin phantoms with a measurable structure and optical properties, which typically consist of the base material, scatterers, and absorbers, can ensure a controllable DRS analysis to prove the validity of the proposed inverse method.

**Figure 3:**
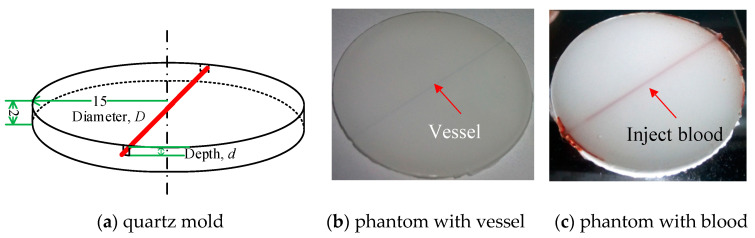
Construction of the skin phantom model.

To prepare a stable, nontoxic phantom with similar optical characteristics to skin tissues—a type of crystal glue—the actual ingredient was epoxy resin (CY186, HUNTSMAN, McIntosh, AL, USA) and medical intralipid (Huarui Pharmaceutical Co., Ltd., Wuxi, China) with 20% concentration were mixed and solidified in a customized mold. The epoxy resin-based material serves as the base media, whereas the fat granule in the intralipid plays the role of light scatterer. Both materials have a limited intrinsic light absorption ability, which is confirmed in the following part. The initial phantom sample was a viscous and milky white liquid titrated with the intralipid until approximately 4% mass fraction was reached. This sample was placed in a thermostatic water bath (approximately 40 °C) and was slowly stirred in one direction for 2 min. Afterward, the homogeneous and bubble-free sample can be finally prepared. Then, the sample was injected into a mold (a quartz glass ring with two U-shaped square holes drilled by laser, as shown in Figure 3a, and the homogeneous skin phantom with controlled thickness (15 mm) and diameter (2 mm) was obtained to measure its optical properties. This cylindrical hole is used to simulate a blood vessel with diameter of 300 μm and depth of 200 μm~1800μm.

To simulate blood vessel, a thin metal wire with a specific depth and diameter was buried into the homogeneous phantom, as indicated by the red line shown in Figure 3a. During the fabrication of the phantom, the wire was ensured to be straight and clinging to the bottom of the U-shaped hole (with a specific diameter and depth), and the machining error was smaller than 20 µm. After injecting the phantom sample, two glass plates were used to clamp the ring tightly to the top and bottom surfaces. After the mold is fixed, the metal wire is unreeved to form a hole and blood is injected to simulate the blood vessel. After approximately 30 h of solidification, the metal wire was slowly pulled out, and a model that contained a tubular channel with a specified diameter was obtained. The discrete vessel skin phantom with known vessel depth and diameter can be obtained by injecting blood into the channel to validate the DRS-based inverse method, as shown in Figure 3b,c.

**Figure 4:**
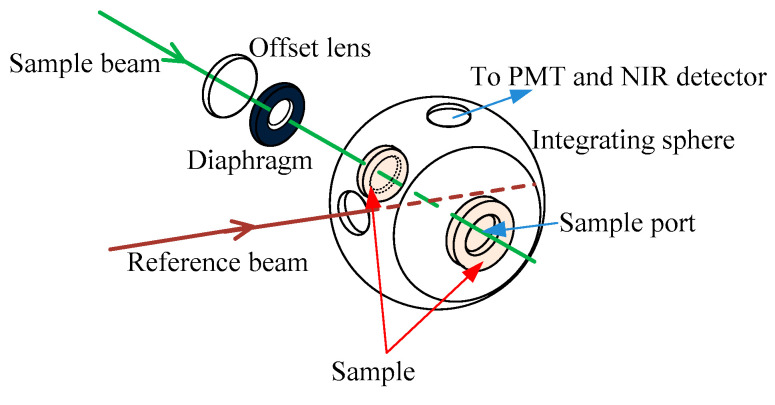
Schematic of spectral measurement.

As illustrated in Figure 4, the spectroscopic data were measured via UV–VIS spectrophotometer (Carry 5000, Agilent Technologies Inc., Santa Clara, CA, USA) that can record data from 190 nm to 3300 nm, with the sample placed in the sample port on the integrating sphere to measure reflectance (at the front port) and transmittance (at the back port).

### 2.3. Overview of the DRS-Based Inverse Method

**Figure 5:**
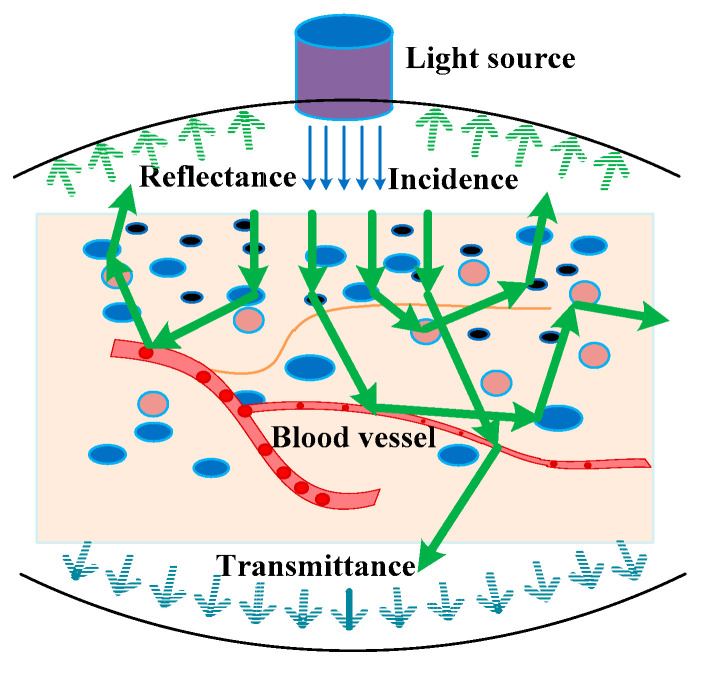
Schematic of light–tissue interaction.

The DRS-based inverse method comprises two steps to obtain the optical and structural parameters of turbid media. The first is to obtain the spectroscopy information, and the second is to construct the relationship between DRS and the optical or structural parameters to be resolved. Figure 5 schematically presents how light escapes from a tissue and forms a spectrum, where particles with various colors indicate different light scatterers, e.g., red blood cells (red), melanin (black), and cell nuclei (blue).

During the interaction between light and tissues, light energy conservation can be expressed by:

(1) $$R+Abs+T_{t}+E_{s}=1$$

**Figure 6:**
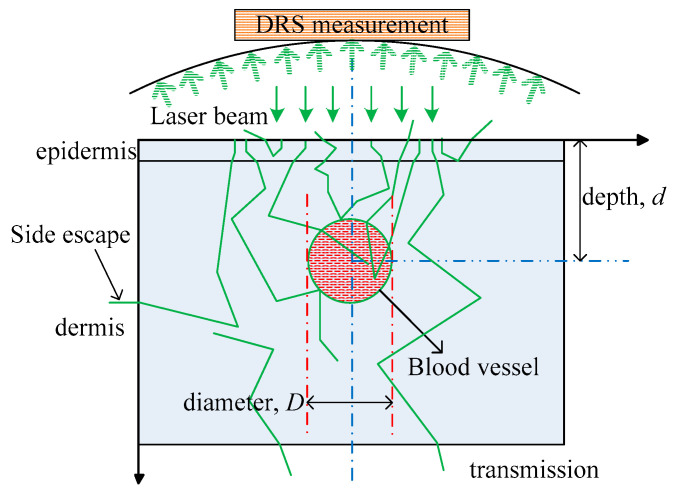
Schematic of DRS measurement.

When the total amount of laser energy is 1; *R* and *Abs* represent the total reflection and total energy deposition within the tissue domain, reflection *R* includes specular scattering and diffuse scattering, and *T* and *E* denote the transmittance and side escape of scattered light, respectively. A schematic of DRS is provided in Figure 6. For a certain wavelength (e.g., the 585 nm pulsed dye laser), the target area, such as a blood vessel, may selectively absorb laser energy. Thus, vessel location (vessel depth) and size (diameter) not only directly influence Abs but also exert a strong influence on photon migration within the entire tissue domain and further affect *R*. In general, *R* exhibits a positive correlation with vessel depth (*d*) and a negative correlation with vessel diameter (*D*). The measured DRS is physically equivalent to *R*_D_, which motivates the verification of the theoretical model by comparing the results of the experiments and the numerical simulations.

In terms of the interaction between laser and skin tissues, the forward problem (to get light distribution within the tissues and the spectral information of the tissue surface with known optical and structural parameters of the laser source and biological tissues) and the inverse problem (to extract optical or structural parameters of the sample medium with known laser sources and surface spectroscopic data) can be described as follows:

(2) $$\begin{array}{l} \left[f_{1}(r),f_{2}(r)\right]=F\left(S,G,p(r)\right)\;\mathrm{Forward}\\ \left[G,p(r)\right]=F^{-1}(S,f_{1}(r))\;\;\;\mathrm{Inverse} \end{array}$$

where *S* and *G* depict the parameters of the laser source and tissue medium, respectively; *f*_1_ is the spectroscopic data and *f*_2_ is the light distribution within tissues; *p* is the optical properties of the tissue, including *μ*_a_, *μ*_s_, and *g*; and **r** is the space vector. **r** indicates the photon’s position vector, with the origin at the laser spot center on the tissue surface. *F* is the general model function, which refers to the theoretical model of light propagation or the experimental model to determine optical properties. As mentioned earlier, the primary dilemma of inverse problems is ill-posedness, i.e., *G* and *p* cannot be obtained simultaneously in Equation (2). Structural parameters can be extracted with the knowledge of optical properties, or vice versa.

**Figure 7:**
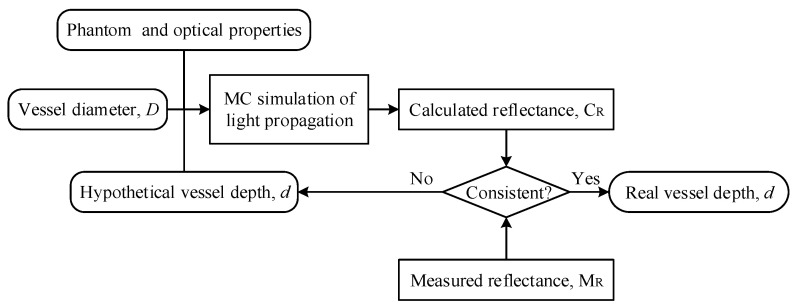
DRS based inverse method for estimating blood vessel depth in the PWS tissue model.

In the PWS tissue model, the diffuse reflectance depends on the reflection spectrum collection method, the skin geometry and physiological/morphological characteristics considered. This article simplified the model, as shown in Ref. [32]. For blood vessels parallel to the skin surface with known optical parameters, the reflection spectrum can be expressed as a function of blood vessel diameter and depth: *R* = *f* (*D*, *d*). In the current study, we assume that *D* is known (e.g., via LSI). Thus, the main task in skin tissue detection is to acquire vessel depth *d*, the extraction of which can follow the technical route in Figure 7. First, the vessel diameter *D* and diffused reflectance *R*_0_ in the tissue region of interest can be obtained through LSI and spectrometry, respectively. Then, MC simulation can be conducted to calculate reflectance *R*_c_ with an assumed vessel depth *d*. The real blood vessel depth can be obtained by adjusting *d* when *R*_0_ is consistent with *R*_c_.

### 2.4. Integrated IAD–FMC Method

In this section, the integrated IAD–FMC method was introduced to extract the optical properties of the sample. The basic concept is to use the preliminary optical properties estimated through IAD by iterating the adding-doubling method with given reflection and transmission as the initial value of FMC to obtain an accurate estimation of the real optical properties. The FMC program used in this study directly complies with our previous work [32].

**Figure 8:**
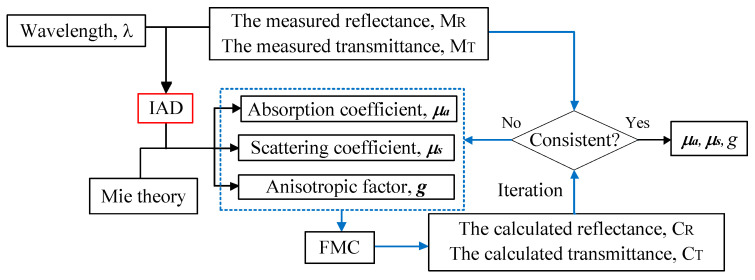
IAD–FMC coupling method.

The procedures of the integrated IAD–FMC method are illustrated in Figure 8. The reflection and transmittance of tissue phantom with known geometric parameters are measured through ultraviolet–visible (UV–VIS) spectroscopy within a wavelength (λ) ranging from 300 nm to 1200 nm. Taking the measured diffuse reflectivity MR and transmissivity MT as inputs of IAD, the corresponding μa and μs can be obtained through IAD, and anisotropic factor g can be obtained on the basis of Mie theory. Reflectivity CR and transmissivity CT can be calculated through FMC. An accurate estimation of the optical properties can be obtained when the calculation matches the measurement.

Good agreement between CR/CT and MR/MT is usually difficult to achieve. Repetitive iterations through FMC are required to optimize the combination of optical properties. μa, μs, and g cannot be simultaneously obtained because excessively many unknowns make the reverse problem to remain unsolved. In IAD, μa and μs are the outputs, and g is set to zero by default. Mie theory can be used to estimate g when particle concentration I, diameter (dp), refractive index of the particle (np), and ambient medium are known because the prepared skin phantom with known optical properties to confirm the validation of the inverse method is a suspension of intralipid granules in an epoxy resin background media. C can be calculated on the basis of the composition proportion of the phantom. dp is roughly estimated to be 0.5 μm. Refractive indexes were measured using an Abbe refractometer. Under these conditions, the calculated g through Mie theory is close to zero (approximately 10^−5^) and exhibits a decreasing trend from visible light to infrared. Thus, the default value of zero is acceptable.

The skin phantom with measurable structure and optical properties can be used in the quantitative verification of IAD–FMC method. A stable, nontoxic phantom with similar optical characteristics to skin tissue was prepared. According to Section 2.2, we make a phantom without blood vessels inside. The initial phantom sample became a viscous and milky white liquid titrated with the intralipid through mixing until approximately 4% mass fraction was reached. This sample was placed in a thermostatic water bath (approximately 40 °C) and slowly stirred in one direction for 2 min. The bubble-free sample was prepared and injected into a mold (quartz glass ring), and the homogeneous skin phantom with controlled thickness (15 mm) and diameter (2 mm) was obtained to measure its optical properties through UV–VIS spectroscopy.

**Figure 9:**
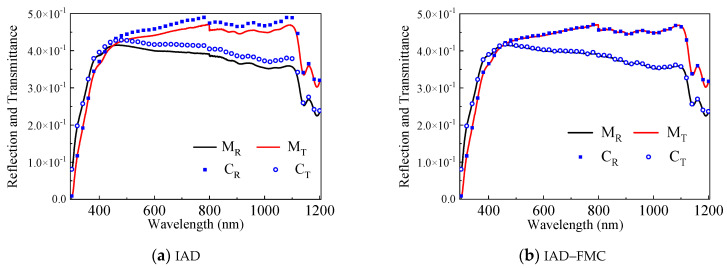
Measured and calculated reflection and transmittance.

**Figure 10:**
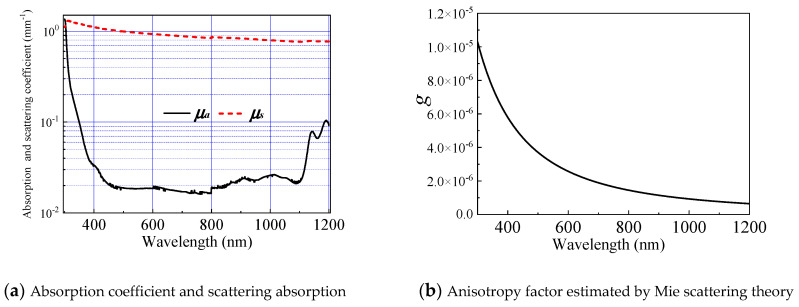
Optical parameters of phantom.

Figure 9 shows the extracted reflection and transmittance of the phantom using IAD and IAD–FMC methods. The maximum error between the calculated and measured spectroscopic data reduces from 10% (IAD only, Figure 9a) to less than 1% using the IAD–FMC method (Figure 9b). The absorption and scattering coefficients are obtained through IAD–FMC method, as shown in Figure 10.

## 3. Extraction of Vessel Depth

**Figure 11:**
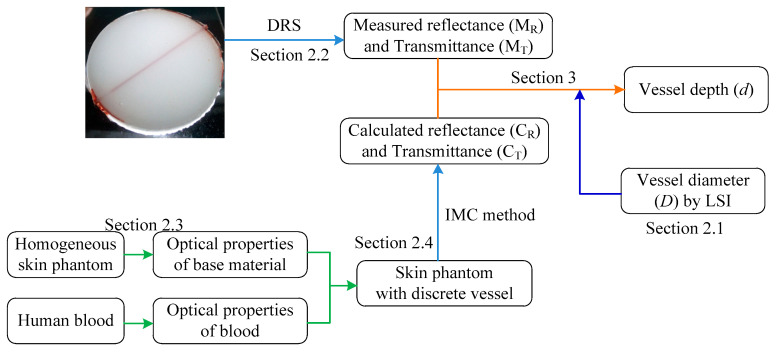
Extraction of vessel depth via the DRS based inverse method in the PWS tissue model.

We have described the process of obtaining basal tissue optical parameters through the IAD–FMC method in Section 2.4. With known optical properties, the structural information of skin tissues can be estimated via the DRS-based inverse method. When vessel diameter is obtained via LSI, only the depth of the target PWS vessel needs to be estimated, thereby overcoming the ill-posedness of the inverse problem. In this section, we extract the depth of the PWS vessel with controllable optical properties and vessel diameter. The validation of this inverse extraction procedure can be demonstrated by the discrete vessel skin phantom in Figure 3, and the technical route is shown in Figure 11. After acquiring the optical properties of the homogeneous phantom material and fresh blood from volunteers (informed consent was obtained), vessel depth can be extracted by comparing the calculated and measured spectroscopic data.

### 3.1. Depth-Related Spectral Signal and Error Propagation

**Figure 12:**
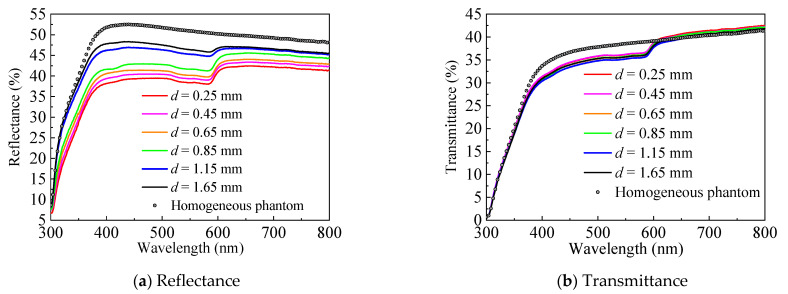
Vessel depth-related spectral signal.

**Figure 13:**
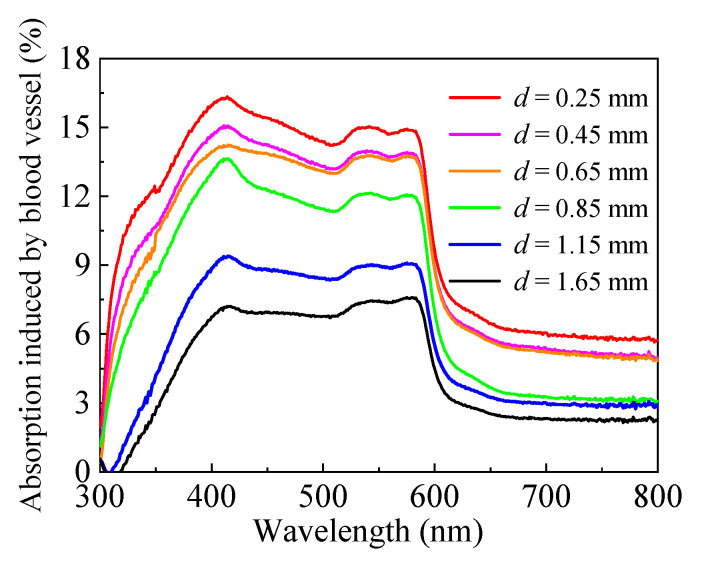
Light absorption induced by the blood vessel with different depths.

This section presents the spectral measurement of the discrete vessel skin phantom with different vessel depths (*d*) (0.25 mm~1.65 mm) and known diameter (*D* = 300 μm). Figure 12 shows the measured reflectance and transmittance within the range of 300 nm to 800 nm. Figure 13 indicates the energy absorption induced by the blood vessel, which was obtained by subtracting the energy absorption by the homogeneous skin phantom without blood vessel. Apparently, the blood vessel can reduce reflectance and transmittance, but only reflectance demonstrates a relatively sensitive dependence on vessel depth (the resolution is approximately 0.7% per 0.1 mm for the current phantom medium). The characteristic reflected by the depth of different blood vessels is the difference in reflectivity. After being scattered and absorbed by the tissue, the transmitted photons are less, and more photons are reflected.

### 3.2. Reconstruction of the Spectral Signal

**Figure 14:**
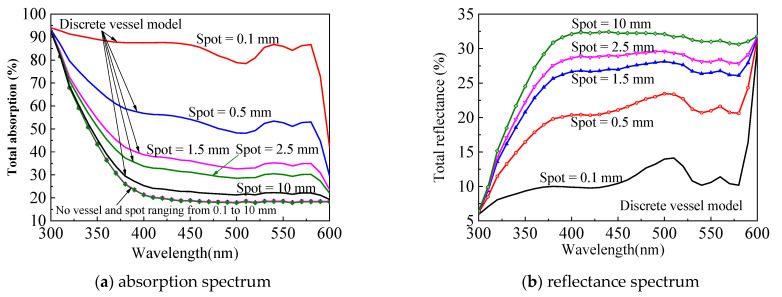
Beam spot-affected spectral signal.

The reconstruction of the spectral signal via MC simulation can facilitate the investigation of spectral–depth relation in a controllable and accurate manner. First, we checked the effect of beam spot on the simulation results within the 300–600 nm band, where blood shows strong hump-like absorption. As shown in Figure 14a, the homogenous skin phantom without blood vessel (dotted lines) will not be affected by the size of beam spot. If a blood vessel exists, however, spot size will seriously influence energy deposition (solid lines in Figure 14a) and spectral signal (Figure 14b). The spectral signal becomes stronger with increasing the spot size, which is beneficial for signal detection but not for the representation of the target blood vessel, because its characteristics are fading away. As shown in Figure 14, the classical hump profile cannot be observed clearly for a spot larger than 2.5 mm. To guarantee a strong signal and distinguished characteristic, an optimal beam spot of 0.5 mm is recommended for the spectral measurement during DRS analysis, which will make the spectral measurement easy to detect and sensitive to vessel distribution. The program used in this study directly complies with our previous work [32].

**Figure 15:**
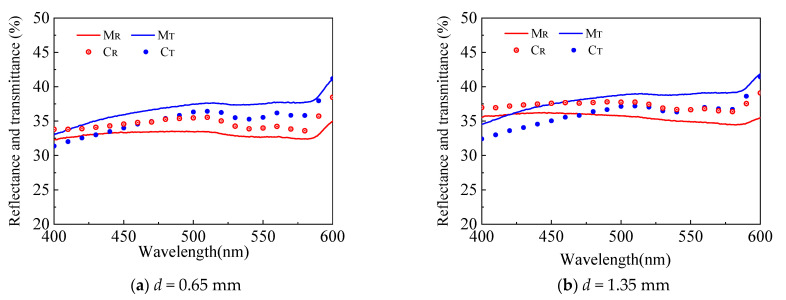
Comparison between the measured and calculated spectroscopic data in the discrete vessel skin phantom with different depths.

**Figure 16:**
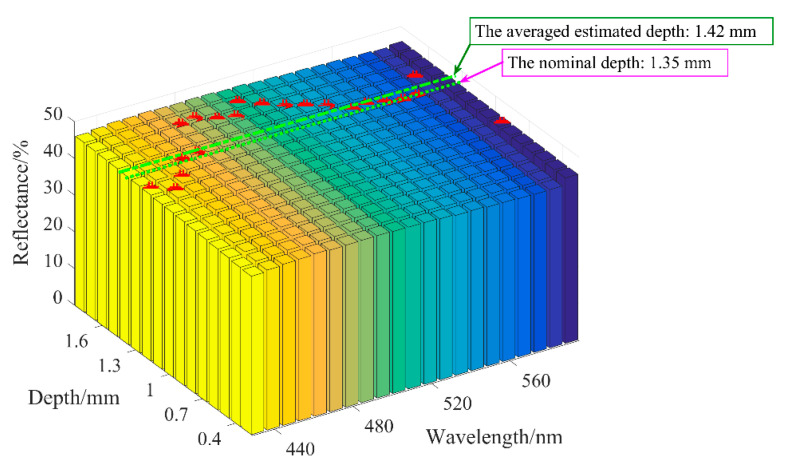
Extraction of vessel depth within the discrete vessel skin phantom and correspondence between blood vessel depth and reflectance spectrum.

With the optimal spot size, the reflectance and transmittance in the blood vessel-embedded skin phantom were measured and compared with the spectral signal reconstructed by MC simulation, as presented in Figure 15. The blood vessel is buried at a depth of 0.65 mm, and the depth of 1.35 mm can also be evaluated by the flip of the phantom. As shown in Figure 16, the reconstructed spectral signal based on MC simulation can accurately characterize the measured signal with an absolute error smaller than 5%.

In Figure 11, vessel depth is the input and spectroscopic data represents the output. To estimate the vessel depth, the input will be the measured spectroscopic data. We can infer that the two processes are interlinked and equivalent, and the extraction procedure is as follows. With the measured reflectance *M*_R_ and the unknown vessel depth (*d*), two predicted values, *d*_1_ and *d*_2_, can be introduced, which ensures that the real vessel depth *d* satisfies *d*_1_ < *d* < *d*_2_ (equivalently, the corresponding calculated reflectance *C*_R1_ and *C*_R2_ satisfy *C*_R2_ < *M*_R_ < *C*_R1_). The accurate vessel depth can be quickly located via the dichotomization of *d*_1_ and *d*_2_. To demonstrate the aforementioned procedure, a discrete vessel skin phantom with a vessel depth of 1.35 mm and a diameter of 0.3 mm was verified. As illustrated in Figure 16, the average relative error of the estimated vessel depth is less than 5%, which verified the extraction of the blood vessel depth in skin tissue via DRS.

**Figure 17:**
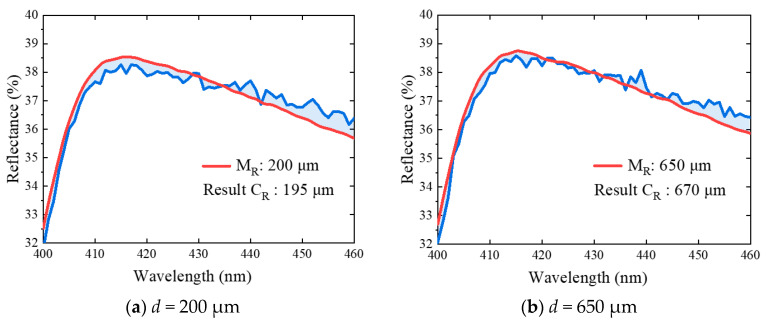

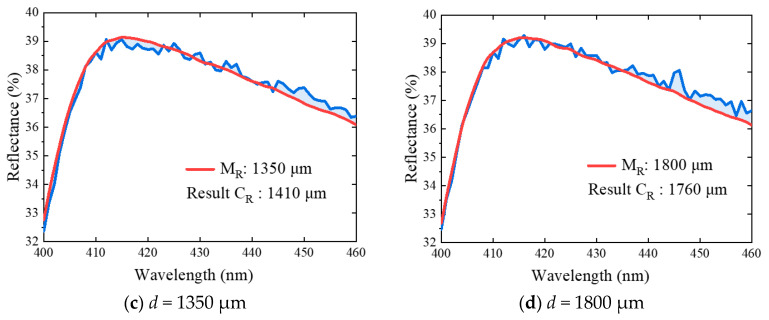
Verification of blood vessel depth extraction (400–460 nm).

Afterwards, the spectra of tissue phantom containing blood vessel with diameter of 100 μm and depth of 200 μm, 650 μm, 1350 μm, and 1800 μm were measured to test the inversed method, as illustrated in Figure 17. Here, we chose a spectral range of 400–600 nm, because it is the absorption peak of hemoglobin and sensitive to vessel depth. The reconstructed spectrum signal based on MC simulation can accurately characterize the measurement signal. Comparing the calculated blood vessel depth with the known depth, the inversion error is less than 5%. The error in calculating the spectrum comes from two aspects: one is the light propagation process, the other is the acquisition of optical parameters. It further verifies the versatility and accuracy of this method in obtaining subcutaneous vascular microstructure information.

## 4. Discussion

This work developed an inverse methodology based on the IAD–FMC method, and diffuse reflectance spectroscopy was established to extract the structural properties of skin tissue, which was experimentally verified by constructing tissue phantom with known blood vessel depth and diameter. Compared with the previous methods of spectral inversion of tissue parameters, the process is complete and systematic. Compared with other methods of blood vessel depth measurement, such as anatomical method [33], diffusion approximation [34], and isobaric wavelength method [35], the present method is more accurate and non-invasive, which is suitable for a blood vessel with depth of 200~1800 µm and high absorption chromophores (red blood cells).

However, when the aforementioned method is applied to real skin, it requires accurate optical parameters of the skin tissue including melanin content, epidermis thickness, absorption, and scattering coefficient. Together with the development of instant acquisition technology of melanin content and other parameters, this study can be applicable to humans in the future.

## 5. Conclusions

In this study, a DRS-based inverse method was developed to extract the structural parameters of skin tissues. The model was experimentally validated by constructing a skin phantom and conducting spectral measurements, which demonstrated consistency between the measured and calculated spectroscopic data. The FMC iteration was accelerated by using the IAD result as initial input.

Taking laser therapy of PWS as an example, blood vessel depth can be extracted by obtaining vessel diameter from LSI. For this case, the optimal spot size is 0.5 mm. Vessel depth extraction was demonstrated in a skin phantom with a single discrete vessel buried in a known depth, where the average relative error is less than 5%. The depth inversion can reach 1800 μm to solve the problem of skin tissue structure detection under a more economical equipment. The theoretical model in this paper realizes the process of retrieving tissue structure, optical information through spectral information, and provides effective theoretical guidance for the clinical diagnosis of PWS and other vascular skin diseases, especially the retrieval of focal microvessel depth information.

## 6. Patents

Inventor: Bin Chen, Yong Zhang, Dong Li. Assignee: Xi’an Jiaotong University. ZL 20181032139.0, publication of 2021.

## Data Availability

Data sharing not applicable.
